# 3,6-Dibromo-7-ethyl­amino-4-methyl-2*H*-chromen-2-one

**DOI:** 10.1107/S160053681201077X

**Published:** 2012-03-17

**Authors:** Ting Zhang, Huai-jie Xing, Chun-bao Miao, Xiao-qiang Sun, Hai-tao Xi

**Affiliations:** a Key Laboratory of Fine Chemical Engineering, Changzhou University, Changzhou 213164, Jiangsu, People’s Republic of China

## Abstract

In title compound, C_12_H_11_Br_2_NO_2_, the coumarin ring system is almost planar, the two rings being inclined to one another by 1.40 (15)°. There are two short intra­molecular inter­actions (N—H⋯Br and C—H⋯Br) involving the Br atoms. In the crystal, mol­ecules stack along the *a*-axis direction *via* π–π inter­actions; the centroid–centroid distances vary from 3.6484 (19) to 3.7942 (19) Å.

## Related literature

For the synthesis of the title compound, see: Belluti *et al.* (2010 ▶). For geometrical details of a coumarin compound, see: Kruszynski *et al.* (2005 ▶).
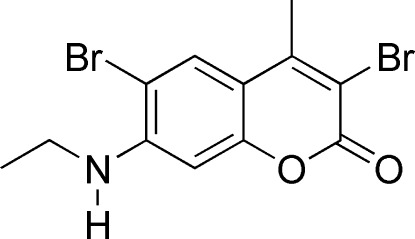


## Experimental

### 

#### Crystal data

C_12_H_11_Br_2_NO_2_
*M* *_r_* = 361.04
Triclinic, 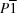

*a* = 7.5795 (9) Å
*b* = 7.6839 (9) Å
*c* = 11.2610 (14) Å
α = 93.628 (2)°
β = 98.288 (3)°
γ = 102.626 (3)°
*V* = 630.24 (13) Å^3^
*Z* = 2
Mo *K*α radiation
μ = 6.42 mm^−1^
*T* = 293 K
0.20 × 0.18 × 0.15 mm

#### Data collection

Enraf–Nonius CAD-4 diffractometer
Absorption correction: ψ scan (North *et al.*, 1968 ▶) *T*_min_ = 0.360, *T*_max_ = 0.446
3658 measured reflections
2314 independent reflections
1928 reflections with *I* > 2σ(*I*)
*R*_int_ = 0.023
3 standard reflections every 200 reflections intensity decay 1%

#### Refinement

*R*[*F* ^2^ > 2σ(*F* ^2^)] = 0.031
*wR*(*F* ^2^) = 0.097
*S* = 1.00
2314 reflections
160 parameters
1 restraint
H atoms treated by a mixture of independent and constrained refinement
Δρ_max_ = 0.51 e Å^−3^
Δρ_min_ = −0.42 e Å^−3^

### 

Data collection: *CAD-4 Software* (Enraf–Nonius, 1985 ▶); cell refinement: *CAD-4 Software*; data reduction: *XCAD4* (Harms & Wocadlo, 1995 ▶); program(s) used to solve structure: *SHELXS97* (Sheldrick, 2008 ▶); program(s) used to refine structure: *SHELXL97* (Sheldrick, 2008 ▶); molecular graphics: *SHELXTL* (Sheldrick, 2008 ▶); software used to prepare material for publication: *SHELXTL*.

## Supplementary Material

Crystal structure: contains datablock(s) I, global. DOI: 10.1107/S160053681201077X/su2385sup1.cif

Structure factors: contains datablock(s) I. DOI: 10.1107/S160053681201077X/su2385Isup2.hkl

Supplementary material file. DOI: 10.1107/S160053681201077X/su2385Isup3.cml

Additional supplementary materials:  crystallographic information; 3D view; checkCIF report

## Figures and Tables

**Table 1:** Hydrogen-bond geometry (Å, °)

*D*—H⋯*A*	*D*—H	H⋯*A*	*D*⋯*A*	*D*—H⋯*A*
N1—H1⋯Br1	0.86 (3)	2.67 (3)	3.055 (3)	109 (2)
C10—H10*A*⋯Br2	0.96	2.68	3.221 (4)	116

**Fig. 1.**
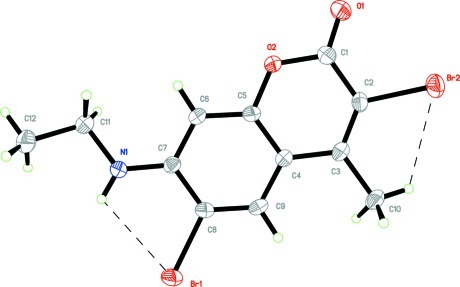
The molecular structure of the title molecule, with the atom-numbering scheme. Displacement ellipsoids are drawn at the 50% probability level. The short Br···H interactions are shown as dashed lines.

**Fig. 2.**
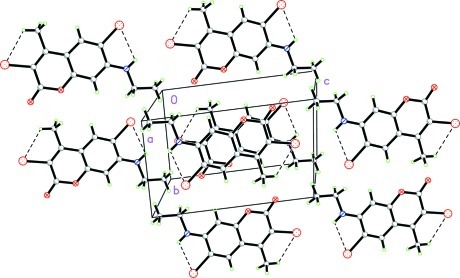
A view along the a axis of the crystal packing of the title compound. The short Br···H interactions are shown as dashed lines.

## supplementary crystallographic information

### Comment

The title compound is used as an important intermediate to synthesis fluorescent tracers, for example, it has been recognized as an effective protein tracer (Belluti *et al.*, 2010). Herein we report on the crystal structure of the title compound, which is illustrated in Fig. 1.

The coumarin ring system is almost planar with a dihedral angle involving rings (O2,C1-C5) and (C4-C9) of only 1.40 (2) °. This is normal for such coumarin compounds (Kruszynski *et al.*, 2005). The bromine atoms are involved in short Br···H interactions (Table 1).

In the crystal, the molecules stack along the a axis direction (Fig. 2). There are a number of π–π interactions present: Cg1···Cg1^i^ 3.7580 (19) Å; Cg2···Cg1^i^ 3.6484(19 Å; Cg2···Cg2^ii^ 3.7942 (19) Å [where Cg1 is the centroid of ring (O2,C1-C5); Cg2 is the centroid of ring (C4-C9); symmetry codes: (i) -x+1, -y+1, -z+1; (ii) -x+2, -y+1, -z+1].

### Experimental

The title compound was prepared by the method reported by (Belluti *et al.*, 2010). To a suspension of 4-methyl-7-*N*,*N*-diethylamino coumarin (5 mmol, 1.61 g) and bromosuccinimide (6 mmol, 1.06 g) in carbon tetrachloride (50 ml), a catalytic amount of benzoyl peroxide was added. The reaction mixture was refluxed for 8 h, then the succinimide produced during the reaction was filtered off. The resulting mixture was washed with water, dried and the solvent was removed under reduced pressure. The pale yellow product obtained was recrystallized from ethanol, yielding colourless block-like crystals of the title compound on evaporating the solvent slowly at room temperature for about 5 days.

### Refinement

The NH H-atom was located in a difference electron-density map and was freely refined. The C-bound H-atoms were included in calculated positions and treated as riding atoms: C-H = 0.93, 0.97 and 0.96 Å for CH, CH_2_ and CH_3_ H-atoms, respectively, with U_iso_(H) = k × U_eq_(parent C-atom), where k = 1.5 for CH_3_ H-atoms and = 1.2 for other H-atoms.

### Crystal data

**Table:**

C_12_H_11_Br_2_NO_2_	*Z* = 2
*M**_r_* = 361.04	*F*(000) = 352
Triclinic, *P*1	*D*_x_ = 1.903 Mg m^−^^3^
Hall symbol: -P 1	Mo *K*α radiation, λ = 0.71073 Å
*a* = 7.5795 (9) Å	Cell parameters from 1771 reflections
*b* = 7.6839 (9) Å	θ = 2.7–26.5°
*c* = 11.2610 (14) Å	µ = 6.42 mm^−^^1^
α = 93.628 (2)°	*T* = 293 K
β = 98.288 (3)°	Block, colourless
γ = 102.626 (3)°	0.20 × 0.18 × 0.15 mm
*V* = 630.24 (13) Å^3^	

### Data collection

**Table:**

Enraf–Nonius CAD-4 diffractometer	1928 reflections with *I* > 2σ(*I*)
Radiation source: fine-focus sealed tube	*R*_int_ = 0.023
Graphite monochromator	θ_max_ = 25.5°, θ_min_ = 1.8°
ω/2θ scans	*h* = −9→8
Absorption correction: ψ scan (North *et al.*, 1968)	*k* = −8→9
*T*_min_ = 0.360, *T*_max_ = 0.446	*l* = −11→13
3658 measured reflections	3 standard reflections every 200 reflections
2314 independent reflections	intensity decay: 1%

### Refinement

**Table:**

Refinement on *F*^2^	Primary atom site location: structure-invariant direct methods
Least-squares matrix: full	Secondary atom site location: difference Fourier map
*R*[*F*^2^ > 2σ(*F*^2^)] = 0.031	Hydrogen site location: inferred from neighbouring sites
*wR*(*F*^2^) = 0.097	H atoms treated by a mixture of independent and constrained refinement
*S* = 1.00	*w* = 1/[σ^2^(*F*_o_^2^) + (0.0633*P*)^2^ + 0.0665*P*] where *P* = (*F*_o_^2^ + 2*F*_c_^2^)/3
2314 reflections	(Δ/σ)_max_ = 0.001
160 parameters	Δρ_max_ = 0.51 e Å^−^^3^
1 restraint	Δρ_min_ = −0.42 e Å^−^^3^

### Special details

**Table:**

Geometry. All e.s.d.'s (except the e.s.d. in the dihedral angle between two l.s. planes) are estimated using the full covariance matrix. The cell e.s.d.'s are taken into account individually in the estimation of e.s.d.'s in distances, angles and torsion angles; correlations between e.s.d.'s in cell parameters are only used when they are defined by crystal symmetry. An approximate (isotropic) treatment of cell e.s.d.'s is used for estimating e.s.d.'s involving l.s. planes.
Refinement. Refinement of *F*^2^ against ALL reflections. The weighted *R*-factor *wR* and goodness of fit *S* are based on *F*^2^, conventional *R*-factors *R* are based on *F*, with *F* set to zero for negative *F*^2^. The threshold expression of *F*^2^ > σ(*F*^2^) is used only for calculating *R*-factors(gt) *etc*. and is not relevant to the choice of reflections for refinement. *R*-factors based on *F*^2^ are statistically about twice as large as those based on *F*, and *R*- factors based on ALL data will be even larger.

### Fractional atomic coordinates and isotropic or equivalent isotropic displacement parameters (Å^2^)

**Table:**

	*x*	*y*	*z*	*U*_iso_*/*U*_eq_	
Br1	0.93608 (5)	0.72412 (5)	0.21480 (3)	0.05088 (16)	
Br2	0.64459 (6)	0.54212 (6)	0.87739 (3)	0.05501 (16)	
O1	0.5330 (4)	0.1746 (4)	0.7375 (2)	0.0561 (7)	
O2	0.6172 (3)	0.2336 (3)	0.5627 (2)	0.0410 (6)	
N1	0.7998 (4)	0.3167 (4)	0.1806 (3)	0.0403 (7)	
C1	0.6005 (5)	0.2886 (5)	0.6787 (3)	0.0414 (8)	
C2	0.6637 (5)	0.4800 (5)	0.7146 (3)	0.0388 (7)	
C3	0.7310 (4)	0.6008 (4)	0.6420 (3)	0.0358 (7)	
C4	0.7489 (4)	0.5355 (4)	0.5224 (3)	0.0335 (7)	
C5	0.6917 (4)	0.3514 (4)	0.4864 (3)	0.0334 (7)	
C6	0.7056 (4)	0.2766 (4)	0.3743 (3)	0.0352 (7)	
H6	0.6655	0.1534	0.3547	0.042*	
C7	0.7792 (4)	0.3845 (4)	0.2908 (3)	0.0331 (7)	
C8	0.8355 (4)	0.5712 (4)	0.3261 (3)	0.0355 (7)	
C9	0.8197 (4)	0.6442 (4)	0.4371 (3)	0.0358 (7)	
H9	0.8565	0.7677	0.4561	0.043*	
C10	0.7902 (5)	0.7994 (5)	0.6800 (3)	0.0480 (9)	
H10A	0.7510	0.8247	0.7552	0.072*	
H10B	0.7361	0.8632	0.6195	0.072*	
H10C	0.9213	0.8366	0.6894	0.072*	
C11	0.7252 (5)	0.1281 (5)	0.1345 (3)	0.0442 (8)	
H11A	0.5925	0.1010	0.1252	0.053*	
H11B	0.7695	0.0515	0.1913	0.053*	
C12	0.7831 (6)	0.0925 (6)	0.0148 (4)	0.0589 (10)	
H12A	0.9142	0.1137	0.0251	0.088*	
H12B	0.7420	0.1709	−0.0405	0.088*	
H12C	0.7300	−0.0298	−0.0166	0.088*	
H1	0.848 (4)	0.380 (4)	0.128 (2)	0.036 (9)*	

### Atomic displacement parameters (Å^2^)

**Table:**

	*U*^11^	*U*^22^	*U*^33^	*U*^12^	*U*^13^	*U*^23^
Br1	0.0641 (3)	0.0348 (2)	0.0574 (3)	0.00657 (16)	0.02514 (19)	0.01385 (16)
Br2	0.0732 (3)	0.0580 (3)	0.0363 (2)	0.0195 (2)	0.01301 (18)	−0.00175 (17)
O1	0.0735 (18)	0.0491 (16)	0.0446 (15)	0.0014 (13)	0.0231 (13)	0.0100 (12)
O2	0.0551 (14)	0.0292 (11)	0.0361 (13)	0.0006 (10)	0.0133 (11)	0.0011 (9)
N1	0.0510 (17)	0.0316 (14)	0.0416 (16)	0.0079 (12)	0.0197 (14)	0.0049 (12)
C1	0.0445 (18)	0.0410 (19)	0.0381 (19)	0.0082 (15)	0.0064 (15)	0.0061 (15)
C2	0.0437 (18)	0.0423 (18)	0.0297 (17)	0.0106 (14)	0.0041 (14)	−0.0011 (14)
C3	0.0338 (16)	0.0327 (16)	0.0396 (18)	0.0092 (13)	0.0017 (14)	−0.0031 (13)
C4	0.0334 (15)	0.0308 (16)	0.0348 (17)	0.0063 (12)	0.0039 (13)	0.0004 (13)
C5	0.0351 (16)	0.0281 (15)	0.0368 (17)	0.0067 (12)	0.0048 (13)	0.0057 (13)
C6	0.0406 (17)	0.0258 (15)	0.0386 (18)	0.0070 (12)	0.0067 (14)	0.0013 (13)
C7	0.0320 (15)	0.0285 (15)	0.0394 (18)	0.0078 (12)	0.0072 (13)	0.0014 (13)
C8	0.0366 (16)	0.0303 (16)	0.0389 (18)	0.0045 (12)	0.0067 (14)	0.0089 (13)
C9	0.0365 (16)	0.0230 (14)	0.0457 (19)	0.0050 (12)	0.0028 (14)	0.0016 (13)
C10	0.056 (2)	0.0335 (18)	0.050 (2)	0.0035 (16)	0.0098 (17)	−0.0084 (16)
C11	0.051 (2)	0.0350 (18)	0.045 (2)	0.0058 (15)	0.0098 (16)	−0.0020 (15)
C12	0.077 (3)	0.052 (2)	0.047 (2)	0.013 (2)	0.015 (2)	−0.0055 (18)

### Geometric parameters (Å, º)

**Table:**

Br1—C8	1.899 (3)	C6—C7	1.390 (5)
Br2—C2	1.899 (3)	C6—H6	0.9300
O1—C1	1.200 (4)	C7—C8	1.418 (4)
O2—C5	1.375 (4)	C8—C9	1.369 (5)
O2—C1	1.379 (4)	C9—H9	0.9300
N1—C7	1.358 (4)	C10—H10A	0.9600
N1—C11	1.467 (4)	C10—H10B	0.9600
N1—H1	0.857 (5)	C10—H10C	0.9600
C1—C2	1.455 (5)	C11—C12	1.503 (5)
C2—C3	1.342 (5)	C11—H11A	0.9700
C3—C4	1.443 (5)	C11—H11B	0.9700
C3—C10	1.508 (4)	C12—H12A	0.9600
C4—C9	1.401 (5)	C12—H12B	0.9600
C4—C5	1.401 (4)	C12—H12C	0.9600
C5—C6	1.381 (5)		
			
C5—O2—C1	122.3 (3)	C9—C8—C7	122.4 (3)
C7—N1—C11	122.7 (3)	C9—C8—Br1	119.2 (2)
C7—N1—H1	124 (2)	C7—C8—Br1	118.4 (2)
C11—N1—H1	113 (2)	C8—C9—C4	120.9 (3)
O1—C1—O2	116.8 (3)	C8—C9—H9	119.5
O1—C1—C2	127.6 (3)	C4—C9—H9	119.5
O2—C1—C2	115.7 (3)	C3—C10—H10A	109.5
C3—C2—C1	124.2 (3)	C3—C10—H10B	109.5
C3—C2—Br2	123.0 (3)	H10A—C10—H10B	109.5
C1—C2—Br2	112.8 (2)	C3—C10—H10C	109.5
C2—C3—C4	117.8 (3)	H10A—C10—H10C	109.5
C2—C3—C10	123.2 (3)	H10B—C10—H10C	109.5
C4—C3—C10	119.0 (3)	N1—C11—C12	109.7 (3)
C9—C4—C5	116.4 (3)	N1—C11—H11A	109.7
C9—C4—C3	124.5 (3)	C12—C11—H11A	109.7
C5—C4—C3	119.0 (3)	N1—C11—H11B	109.7
O2—C5—C6	115.9 (3)	C12—C11—H11B	109.7
O2—C5—C4	121.0 (3)	H11A—C11—H11B	108.2
C6—C5—C4	123.1 (3)	C11—C12—H12A	109.5
C5—C6—C7	120.3 (3)	C11—C12—H12B	109.5
C5—C6—H6	119.9	H12A—C12—H12B	109.5
C7—C6—H6	119.9	C11—C12—H12C	109.5
N1—C7—C6	122.4 (3)	H12A—C12—H12C	109.5
N1—C7—C8	120.7 (3)	H12B—C12—H12C	109.5
C6—C7—C8	116.9 (3)		
			
C5—O2—C1—O1	179.8 (3)	C9—C4—C5—C6	1.1 (5)
C5—O2—C1—C2	1.1 (4)	C3—C4—C5—C6	−179.2 (3)
O1—C1—C2—C3	−177.1 (4)	O2—C5—C6—C7	−179.9 (3)
O2—C1—C2—C3	1.5 (5)	C4—C5—C6—C7	0.1 (5)
O1—C1—C2—Br2	3.8 (5)	C11—N1—C7—C6	8.0 (5)
O2—C1—C2—Br2	−177.6 (2)	C11—N1—C7—C8	−172.8 (3)
C1—C2—C3—C4	−2.8 (5)	C5—C6—C7—N1	178.4 (3)
Br2—C2—C3—C4	176.3 (2)	C5—C6—C7—C8	−0.8 (5)
C1—C2—C3—C10	178.0 (3)	N1—C7—C8—C9	−178.9 (3)
Br2—C2—C3—C10	−3.0 (5)	C6—C7—C8—C9	0.2 (5)
C2—C3—C4—C9	−178.8 (3)	N1—C7—C8—Br1	1.1 (4)
C10—C3—C4—C9	0.5 (5)	C6—C7—C8—Br1	−179.8 (2)
C2—C3—C4—C5	1.6 (5)	C7—C8—C9—C4	1.0 (5)
C10—C3—C4—C5	−179.2 (3)	Br1—C8—C9—C4	−179.0 (2)
C1—O2—C5—C6	177.8 (3)	C5—C4—C9—C8	−1.7 (5)
C1—O2—C5—C4	−2.2 (5)	C3—C4—C9—C8	178.7 (3)
C9—C4—C5—O2	−178.8 (3)	C7—N1—C11—C12	−175.8 (3)
C3—C4—C5—O2	0.8 (5)		

### Hydrogen-bond geometry (Å, º)

**Table:**

*D*—H···*A*	*D*—H	H···*A*	*D*···*A*	*D*—H···*A*
N1—H1···Br1	0.86 (3)	2.67 (3)	3.055 (3)	109 (2)
C10—H10*A*···Br2	0.96	2.68	3.221 (4)	116
